# *P25* Gene Knockout Contributes to Human Epidermal Growth Factor Production in Transgenic Silkworms

**DOI:** 10.3390/ijms22052709

**Published:** 2021-03-08

**Authors:** Meiyu Wu, Jinghua Ruan, Xiaogang Ye, Shuo Zhao, Xiaoli Tang, Xiaoxiao Wang, Huiping Li, Boxiong Zhong

**Affiliations:** College of Animal Science, Zhejiang University, Hangzhou 310058, China; 11617008@zju.edu.cn (M.W.); jinghuaryan@zju.edu.cn (J.R.); 11817001@zju.edu.cn (X.Y.); asd8899176@163.com (S.Z.); 21717407@zju.edu.cn (X.T.); 21717412@zju.edu.cn (X.W.); 21717409@zju.edu.cn (H.L.)

**Keywords:** transgenic silkworm, transgene, *P25* gene knockout, human epidermal growth factor

## Abstract

Transgenic silkworm expression systems have been applied for producing various recombinant proteins. Knocking out or downregulating an endogenous silk protein is considered a viable strategy for improving the ability of transgenic expression systems to produce exogenous proteins. Here, we report the expression of human epidermal growth factor (hEGF) in a *P25* gene knockout silkworm. The *hEGF* gene regulated by the *P25* gene promoter was integrated into a silkworm’s genome. Five transgenic positive silkworm lineages were generated with different insertion sites on silkworm chromosomes and the ability to synthesize and secrete proteins into cocoons. Then, a cross-strategy was used to produce transgenic silkworms with a *P25* gene knockout background. The results of the protein analysis showed that the loss of an endogenous P25 protein can increase the hEGF production to about 2.2-fold more than normal silkworms. Compared to those of transgenic silkworms with wild type (non-knockout) background, the morphology and secondary structure of cocoon silks were barely changed in transgenic silkworms with a *P25* gene knockout background, indicating their similar physical properties of cocoon silks. In conclusion, *P25* gene knockout silkworms may become an efficient bioreactor for the production of exogenous proteins and a promising tool for producing various protein-containing silk biomaterials.

## 1. Introduction

Basic research aimed at developing clinical treatments for diseases has led to the production of a variety of medically useful proteins, including vaccines, growth factors, hormones, enzymes, and functional protein-based biomaterials (which have recently become a research focus). With the increasing demand for recombinant proteins, and continued discovery of novel medically useful proteins, the improvement of production technology has become a focus of research exploration. Various excellent expression systems based on different hosts have gradually become the primary tools for protein production, including plant [1], *Escherichia coli* (*E. coli*) [2], insect [3], yeast [4], and mammals [5].

Transgenic silkworm bioreactors were established in 2000, when transgenic silkworms were successfully obtained using *piggy*Bac transposon technology [6], and have rapidly developed since then. The silkworm *Bombyx mori*, an important economic holometabolous lepidopteran insect, has been domesticated for thousands of years. Silk, which mainly consists of silk fibroin and sericin, is the main economically important product of the silkworm. Fibroin is synthesized in the posterior silk glands. Its major components include a fibroin heavy chain (FibH), fibroin light chain (FibL), and P25/fibrohexamerin [7]. Sericin is synthesized and secreted by the middle silk gland (MSG) and covers fibroins, binding them together. *FibH*, *FibL*, and *Ser1* gene promoters have been used to express a variety of proteins, including spider silk [8], feline interferon [9], human serum albumin [10], antibodies [11], and collagen [12], due to their strong activity. The *P25* gene promoter is considered to have dual characteristics of a eukaryotic promoter and a silk gland specific promoter [13] and has been proven capable of driving the production of foreign proteins such as human insulin-like growth factor (hIGF-I) [14] and recombinant globulin [15] in silkworms.

To date, the yields of exogenous proteins in transgenic silkworms have not been very impressive. Since silk glands mainly produce silk, most of the materials and energy obtained by silkworms are used for the synthesis of endogenous silk proteins, resulting in the inhibition of exogenous protein expression [16]. Therefore, knocking out or downregulating an endogenous silk protein is considered a viable strategy for increasing the expression of exogenous proteins. The removal of the endogenous FibH protein by zinc finger nuclease (ZFN) technology increased exogenous recombinant protein production [16], demonstrating the feasibility of the above strategy.

Human epidermal growth factor (hEGF) is a single-chain, small acidic polypeptide composed of 53 amino acids. Its molecular weight is approximately 6.2 kD. hEGF is present in various tissues and fluids of the human body. It activates several downstream signaling pathways by binding to the epidermal growth factor receptor (EGFR) [17,18,19]. Since hEGF can strongly promote the proliferation of embryonic cells, skin cells, endothelial cells, and fibroblasts, it has attracted increasing attention in the fields of ulcer treatment and skin wound healing [20]. In this study, we report the expression of hEGF in *P25* gene knockout silkworms. Our results show that knockouts of the endogenous P25 protein can enhance the expression of hEGF under control of the *P25* gene promoter.

## 2. Results

### 2.1. Transgenic Vector Construction and Generation of Transgenic Silkworms

**Figure 1:**
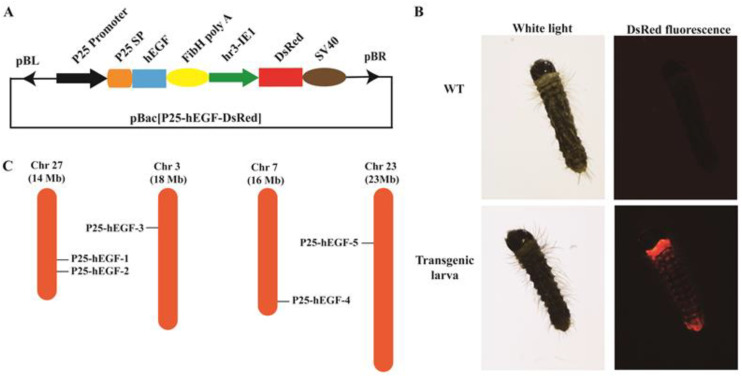
Transgenic vector construction. (**A**) Schematic map of the transgenic vector pBac(P25-hEGF–IE1–DsRed). pBL and pBR: sequences of the left and right arms of the *piggy*Bac transposon, P25 promoter: the promoter sequence of the *P25* gene, *P25* SP: sequence of the *P25* gene signal peptide, *hEGF*: the *hEGF* coding sequence, *FibH* polyA: the polyA signal sequence of the fibroin heavy-chain gene, *hr3-IE1*: combination of the *hr3* enhancer and *IE1* promoter to specific drive marker gene expressions in various tissues and organs of silkworms during different developmental stages, *DsRed*: red fluorescence protein gene, and SV40: the SV40 signal sequence. (**B**) Screening for positive transgenic silkworms by red fluorescence detection. (**C**) Chromosomal transgene insertion sites in five transgenic lines of *Bombyx mori*.

The *piggy*Bac-based (pBac) transgenic vector pBac(P25-hEGF–IE1–DsRed) was constructed to express hEGF in the posterior silk glands (PSGs) of silkworms under the control of the *P25* gene promoter (Figure 1A). A red fluorescence protein (*DsRed*)gene expression driven by the housekeeping *IE1* (immediately early gene 1) promoter was used as a marker. A total of 800 *Lan 10* eggs were microinjected, 103 of which were hatched (Table 1). Transgene-positive newly hatched G1 larvae were identified by fluorescence microscopy (Figure 1B). Five positive broods containing 53-positive individuals were identified, giving a transgenic efficiency of around 14.29% (Table 1). G1-positive broods were reared and sib-mated. Five positive lineages were selected in G2 and named P25-hEGF-1~5.

### 2.2. Gene Integration Site Detection and hEGF Protein Analysis in Transgenic Silkworms

Inverse PCR results showed that, for each of the five selected lineages, a single copy of the *hEGF* transgene was inserted into a unique site in the silkworm genome (Figure 1C). Specifically, P25-hEGF-1 and P25-hEGF-2 shared *hEGF* gene insertion into introns on chromosome 27, P25-hEGF-3 contained an insertion into an intron on chromosome 3, and P25-hEGF-4 harbored an insertion into a gene interval on chromosome 7. Only P25-hEGF-5 exhibited insertion into an exon on chromosome 23. These results confirmed that the *hEGF* gene was successfully integrated into the silkworm genome and that the insertion site was random.

**Figure 2:**
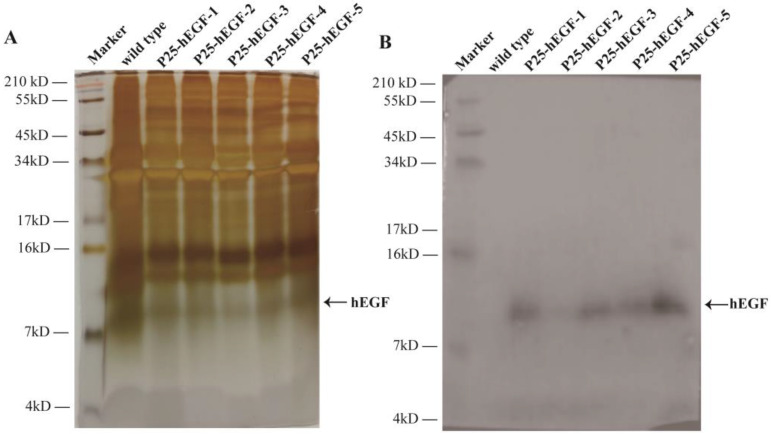
Expression analysis of the hEGF protein in transgenic cocoons. (**A**) SDS-PAGE analysis of the hEGF protein. Marker: protein marker and wild type: protein sample from wild-type cocoons. P25-hEGF-1, -2, -3, -4, and -5: protein samples from transgenic lineages P25-hEGF-1, -2, -3, -4, and -5, respectively. (**B**) Western blot analysis of hEGF protein by using an hEGF antibody.

To confirm hEGF protein expression in transgenic silkworm cocoons, proteins extracted from the cocoon shells from each of the five transgenic lineages, P25-hEGF-1~5, and wild-type *Lan 10* were subjected to SDS-PAGE and Western blotting. Protein bands with a molecular weight of approximately 8 kDa (a little higher than the predicted 6.2 kDa) appeared in transgenic group lines but not in *Lan 10* (Figure 2B). The above results proved that hEGF can be expressed in transgenic silkworms and secreted into transgenic silkworm cocoon shells.

### 2.3. Production of Transgenic Silkworms with a P25 Gene Knockout Background

**Figure 3:**
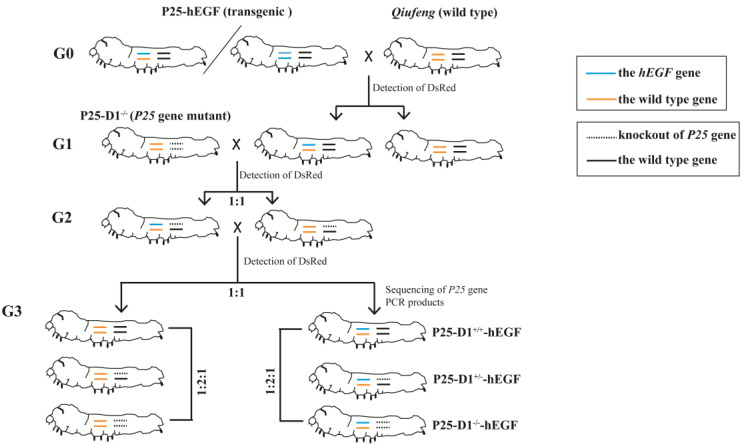
Schematic diagram of the cross-strategy for generating transgenic silkworms with a *P25* gene mutant genetic background. Generation zero (G0)-positive individuals were either heterozygous or homozygous.

**Figure 4:**
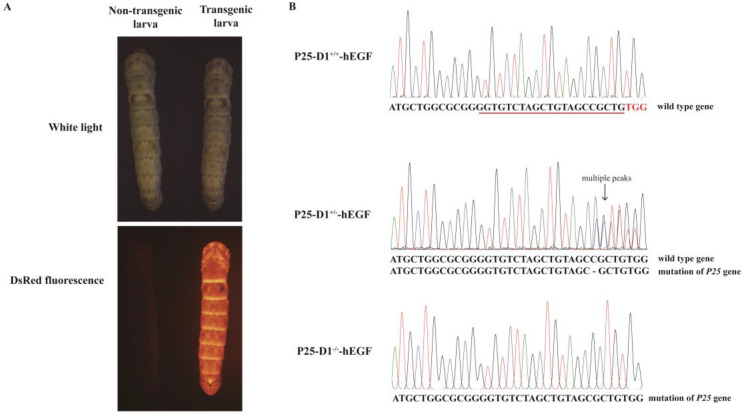
Production of transgenic silkworms in a *P25* gene knockout background. (**A**) Screening for positive transgenic silkworms by red fluorescence detection. (**B**) Sequencing of PCR products: sequencing of silkworm exuviation genomic DNA to detect the genotype at the P25 locus. The sequence underlined in red in the P25-D1^+/+^-hEGF group (upper) was the target site of sgRNA (small guide RNA) for CRISPR/Cas9 (Clustered regularly interspaced short palindromic repeats/an associated protein (Cas9)) editing in our previous research, and the three bases TGG in red was the protospacer adjacent motif (PAM) sequence for Cas9 recognition. The multiple chromatogram peaks at the region flanking the PAM sequence in the P25-D1^+/−^-hEGF group (middle) suggests the successful generation of a heterozygous *P25* gene mutant (black hyphen in the sequence represents the deleted base). The single peak in the chromatogram with a different sequence from wild type at the region flanking the PAM sequence in the P25-D1^−/−^-hEGF group (bottom) suggests the generation of the homozygous *P25* gene mutant.

Silk glands developed normally in P25 knockout silkworms, and their ability to spin silks was like that of wild-type *Qiufeng*, suggesting that the ability of mutants to synthesize and secrete proteins was not affected. To investigate the influence of P25 protein loss on the expression of exogenous proteins, wild-type individuals and transgenic individuals homozygous or heterozygous for *P25* gene knockout were prepared via a cross-strategy (Figure 3). The presence of DsRed fluorescence in larvae indicated the presence of the exogenous *hEGF* gene (Figure 4A). Since there was no significant phenotypic difference at any developmental stage between the *P25* gene knockout and wild-type silkworms, PCR product sequencing of each silkworm’s exuviation genomic DNA was used to determine each individual’s P25 genotype. The chromatogram peaks in the region flanking the protospacer adjacent motif (PAM) sequence (TGG) identified the homozygous and heterozygous *P25* gene knockout individuals (Figure 4B). Three new transgenic lineages with homozygous, heterozygous, and wild-type genetic backgrounds were established and named P25-D1^−/−^-hEGF, P25-D1^+/−^-hEGF, and P25-D1^+/+^-hEGF, respectively.

### 2.4. Phenotype and Protein Analysis of Transgenic Cocoons in a P25 Gene Knockout Background

**Figure 5:**
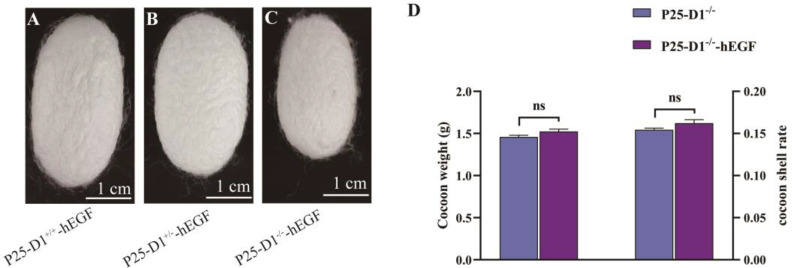
Phenotype and protein analysis of transgenic cocoons in a *P25* gene knockout background. (**A**–**C**) Cocoon phenotypes of transgenic P25-D1^+/+^-hEGF (**A**), P25-D1^+/−^-hEGF (**B**), and P25-D1^−/−^-hEGF (**C**). (**D**) Cocoon weights and cocoon shell rates of non-transgenic P25-D1^−/−^ and transgenic P25-D1^−/−^-hEGF. ns: no significant difference (*n* = 18). The scale bars represent 1 cm.

**Figure 6:**
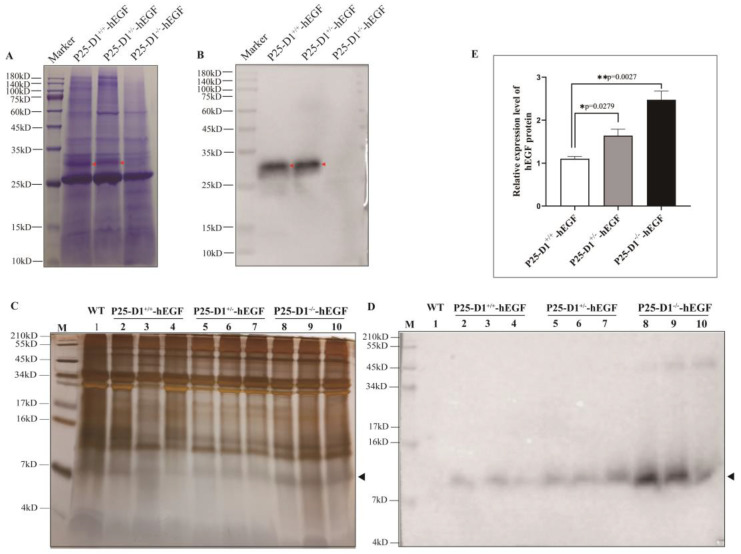
Analysis of hEGF protein expression in hEGF transgenic cocoons in a *P25* gene knockout background. (**A**,**B**) SDS-PAGE (**A**) and Western blotting (**B**) analysis of P25 protein using 15% gradient SDS-PAGE gels. Marker: protein marker and P25-D1^+/+^-hEGF, P25-D1^+/−^-hEGF, and P25-D1^−/−^-hEGF, protein samples from the transgenic P25-D1^+/+^-hEGF, P25-D1^+/−^-hEGF, and P25-D1^−/−^-hEGF groups. The red arrowhead points to the P25 protein. (**C**,**D**) SDS-PAGE (**C**) and Western blotting (**D**) analysis of hEGF protein using 15.5% gradient Tricine-SDS-PAGE gels (Sangon, Shanghai, China). M: protein marker; WT: protein sample from non-transgenic wild-type cocoons; 2–4, 5–7, and 8–10, protein samples in triplicate from the P25-D1^+/+^-hEGF, P25-D1^+/−^-hEGF, and P25-D1^−/−^-hEGF groups, respectively. The black arrowhead points to the hEGF protein. (**E**) Quantitation of hEGF protein bands by using ImageJ software. P25-D1^−/−^-hEGF and P25-D1^−/−^-hEGF produced about 2.2- and 1.5-fold more hEGF, respectively, than P25-D1^+/+^-hEGF (* *p* < 0.05 and ** *p* < 0.01; *n* = 3).

Normal cocoons were successfully harvested from each transgenic group. Compared with heterozygote P25-D1^+/−^-hEGF and wild-type P25-D1^+/+^-hEGF cocoons, homozygous P25-D1^−/−^-hEGF cocoons were smaller (Figure 5A–C). Compared with non-transgenic P25-D1^−/−^ cocoons, the cocoons of transgenic P25-D1^−/−^-hEGF silkworms showed no significant difference in weigh and shell rate (Figure 5D). To further confirm the P25 knockout background, we attempted to detect the P25 protein. Coomassie brilliant blue staining and immunoblotting with an anti-P25 antibody showed that the P25 protein disappeared in the P25-D1^−/−^-hEGF group but remained in the P25-D1^+/+^-hEGF and P25-D1^+/−^-hEGF groups, suggesting the successful P25 knockout in P25-D1^−/−^-hEGF (Figure 6A,B). To confirm the exogenous hEGF expression in P25 knockout silkworms, proteins were solubilized from cocoon layers, separated by SDS-PAGE, and probed with an anti-EGF antibody. Protein bands with a molecular weight of approximately 8 kDa appear in the three transgenic groups: P25-D1^−/−^-hEGF, P25-D1^+/−^-hEGF, and P25-D1^+/+^-hEGF but not in the non-transgenic wild type (Figure 6C,D). To further analyze whether the loss of endogenous P25 would increase the expression of an exogenous protein, hEGF bands were analyzed with ImageJ software. Band densities were normalized against a cocoon. The result showed that P25-D1^−/−^-hEGF and P25-D1^+/−^-hEGF produced about 2.2- and 1.5-fold more protein of the total cocoon than P25-D1^+/+^-hEGF, respectively (Figure 6E), and the differences were statistically significant.

### 2.5. Characteristics of P25-D1^−/−^-hEGF and P25-D1^+/+^-hEGF Cocoon Silks

**Figure 7:**
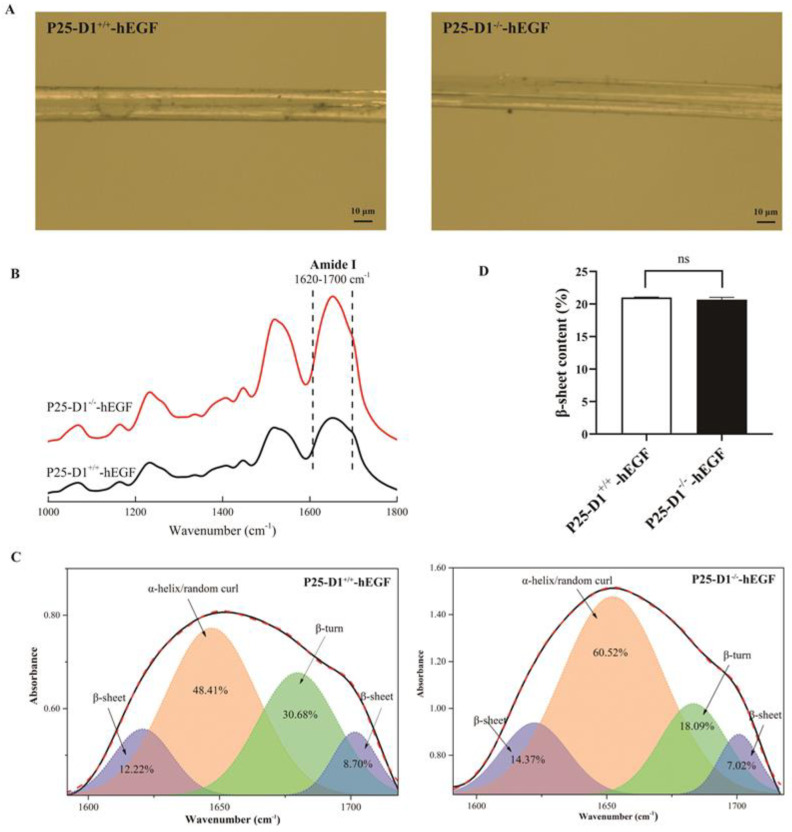
Characteristics of the P25-D1^+/+^-hEGF and P25-D1^−/−^-hEGF cocoon silks. (**A**) Photos on the surfaces of the silk fibers of P25-D1^+/+^-hEGF and P25-D1^−/−^-hEGF. (**B**) Fourier-transform infrared spectroscopy (FTIR) spectrum from 1000–1800 cm^−1^ of the P25-D1^+/+^-hEGF and P25-D1^−/−^-hEGF cocoon silks. (**C**) Deconvolution results of amide I bands from the FTIR spectra of the P25-D1^+/+^-hEGF and P25-D1^−/−^-hEGF cocoon silks. The number in each peak represents the content of different conformations. (**D**) β-sheet contents of the P25-D1^+/+^-hEGF and P25-D1^−/−^-hEGF cocoon silks. The scale bars represent 10 μm.

To identify what characteristic difference of cocoon silks would be produced when hEGF was expressed in *P25* gene knockout silkworms, the surfaces of cocoon silk fibers and the secondary structure were analyzed by a digital microscope at 1000× magnification and Fourier-transform infrared (FTIR) microspectroscopy, respectively. The photos showed there were no evident morphological differences on the surfaces of the silk fibers between the P25-D1^−/−^-hEGF and P25-D1^+/+^-hEGF groups (Figure 7A). Since the amide I band is the stable primary amide band that depends upon the secondary structure of the protein’s backbone [21,22], it was selected to be analyzed here. The FTIR spectra of cocoon silks showed the similar amide I band (1620–1700 cm^−1^) in P25-D1^−/−^-hEGF and P25-D1^+/+^-hEGF groups (Figure 7B). After the peak deconvolution of the amide I band, four strong bands at about 1620 cm^−1^ (β-sheet), 1650 cm^−1^ (α-helix/random curl), 1683 cm^−1^ (β-turn), and 1701 cm^−1^ (β-sheet) were generated and analyzed (Figure 7C), and there was no significant change about the β-sheet contents between the P25-D1^−/−^-hEGF and P25-D1^+/+^-hEGF cocoon silks (Figure 7D).

## 3. Discussion

Increasing the protein yield and developing strategies for large-scale production have long been pursued challenges. Genetic modification technology has contributed to the development of many promising protein production strategies since the 1980s [16]. As an economically important insect, the silkworm *Bombyx mori* has been reportedly used to produce numerous recombinant proteins [23], making it a low-cost, high-yield bioreactor. Compared to another highly efficient expression system, *Escherichia coli* (*E. coli*), which produces the target protein mostly in the form of inclusion body and makes protein purification difficult, it is easy to obtain purified protein from a silkworm. The *P25* gene promoter can drive the large high-level expression of P25 glycoprotein in the posterior silk glands [14], and the concentration of P25 mRNA was found to be roughly equal to FibH mRNA in posterior silk gland cells during the intermolt stage [24,25], suggesting strong *BmP25* gene promoter activity. Thus, the *P25* gene promoter was introduced here to drive *hEGF* gene expression in silkworms. We inserted the *hEGF* gene into the silkworm genome by *piggy*Bac transposon to produce transgenic silkworms. Five transgenic lineages (P25-hEGF-1–5) were obtained by screening for red fluorescence. Our inverse PCR results and Western blotting analyses confirmed the presence of hEGF in transgenic silkworms, confirming the successful preparation of transgenic silkworms. It is worth noting that the hEGF protein was found at approximately 8 kDa rather than the theoretical molecular weight of 6.2 kDa. This might indicate post-translational modification of hEGF in the posterior silk glands [14]. In summary, we confirmed the functionality of our in vitro synthesized *P25* gene promoter and the successful production of transgenic silkworms containing an exogenous *hEGF* gene.

As an important giant organ in the silkworm, the silk gland possesses extraordinary protein production ability. Its primary dedication to silk production is thought to repress the expression of exogenous proteins [16]. P25 protein is a component of silk fibroin which makes up 75% of the total silk protein. The concentration of P25 mRNA in the posterior silk gland cells has been found to be roughly equal to FibH mRNA during the intermolt stage [24,25], indicating that the endogenous *P25* gene may strongly contribute to repressing the exogenous protein expression. Hence, the homozygous *P25* gene mutant P25-D1^−/−^ generated in our lab was mated with hEGF transgenic lineages to prepare transgenic silkworms in a P25 knockout background to explore their efficiency of protein expression. The red fluorescence found in larvae and the results of *P25* gene PCR product sequencing demonstrated that transgenic hEGF P25 knockout lines were produced. Compared with non-transgenic P25-D1^−/−^, however, the transgenic P25-D1^−/−^-hEGF showed no significant difference in the cocoon weight and cocoon shell rate, possibly because the yield of hEGF was too low relative to an entire cocoon or cocoon shell. Immunoblotting for hEGF showed that the P25 knockout strain could act as a host for the expression and secretion of exogenous hEGF protein. The quantitation of hEGF protein bands showed that P25-D1^−/−^-hEGF produced the highest amount of hEGF protein, approximately 2.2-fold more than P25-D1^+/+^-hEGF. P25-D1^+/−^-hEGF produced about 1.5-fold more than P25-D1^+/+^-hEGF. These results suggest that the knockout of endogenous silk fibroin P25 might enhance the production of exogenous proteins and that P25-D1^−/−^ has the potential to be an efficient bioreactor.

Compared to P25-D1^+/+^-hEGF, P25-D1^−/−^-hEGF displayed no morphological difference on the surface of cocoon silks, indicating that the loss of the P25 protein barely had any influence on the morphology of cocoon silks. The β-sheet structure was considered as the dominant factor that answered for the physical properties of silks [26]. In our results, the β-sheet conformation contents were similar in the P25-D1^+/+^-hEGF and P25-D1^−/−^-hEGF groups, suggesting the normal physical properties of P25-D1^−/−^-hEGF cocoon silks. However, the actual influence of the loss of the P25 protein on the properties of silks needs to be explored deeper.

Increasing the yields of hEGF (an excellent healing growth factor) has long been a research focus. Our results suggest that the P25 knockout strain could be an excellent bioreactor for hEGF production via the *P25* gene promoter. Loss of the P25 protein did not damage the silk gland’s ability to synthesize and spin proteins but did increase exogenous protein expression by weakening the repressing effect of endogenous P25 protein. Additionally, since mutations in P25 had almost no influence on the synthesis and secretion of silk proteins, the *P25* gene knockout strain could also be a promising tool for preparing various protein-functional silk biomaterials. This needs to be studied further.

## 4. Materials and Methods

### 4.1. Transgene Vector Construction

Our transgenic vector was constructed on the basis of the previously constructed vector pBac(FibH-hEGF–IE1–DsRed) (unpublished). The mature 162-bp sequence for *hEGF* (GenBank Accession No. X04571.1) was optimized for silkworm codon usage bias. The fragment *Xho*I–P25 promoter–P25 signal peptide–*Age*I was synthesized by Genscript (Nanjing, China) and cloned into the vector pBac(FibH-hEGF–IE1–DsRed) using *Xho*I/*Age*I sites, yielding pBac(P25 promoter–P25 signal peptide-hEGF–IE1–DsRed), which we refer to as pBac(P25-hEGF–IE1–DsRed).

### 4.2. Embryo Injection and Screening for Positive Individuals

The Bombyx mori polyvoltine strain with diapause ability, *Lan10*, was selected for microinjections. Embryo injections and screening for positive individuals were performed as previously described [27]. The transgene plasmid pBac(P25-hEGF–DsRed) was mixed with a helper vector at a 1:1 ratio and microinjected into pre-blastoderm generation zero (G0) embryos. Injected eggs were incubated at 25 °C in 90% humidity until larvae hatched. Larvae were carefully bred, then mated with wild-type individuals to produce the G1 generation. Transgene-positive individuals were screened for expression of the *DsRed* marker gene at the larval stage by using a fluorescence microscope (Olympus SZX16, Tokyo, Japan).

### 4.3. Inverse PCR Analysis

Inverse PCR analysis was performed as previously described [28], with some modifications. Briefly, genomic DNA was isolated from MSGs of positive transgenic larvae on the fifth instar day three using a DNA extraction kit (Sangon, Shanghai, China). This DNA was digested with *Sau3A*I at 37 °C for about 2 h, then circularized by incubating with T4 DNA ligase (TaKaRa, Dalian, China) overnight at 16 °C. Inverse PCR amplification was carried out using the circularized fragments as templates under standard conditions, with primers complementary to the left or right arm of the *piggy*Bac vector. The primers were as follows: pBacR1-F/pBacR1-R and pBacL1-F/pBacL1-R for the first PCR and pBacR2-F/pBacR2-R and pBacL2-F/pBacL2-R for the second PCR (Table 2). The amplification conditions for the first PCR were as follows: 94 °C for 3 min, 35 cycles at 94 °C for 30 s, 49 °C (for pBacR1-F/pBacR1-R) or 56 °C (for pBacL1-F/pBacL1-R) for 30 s, 72 °C for 3 min, and a final extension period of 72 °C for 10 min. Amplification conditions for the second PCR were the same, except that annealing was carried out at 52 °C (for pBacR2-F and pBacR2-R) and 58 °C (for pBacL2-F and pBacL2-R). Amplified fragments were sequenced after cloning into the pMD19-T vector. Searches of the silkworm genome database (https://sgp.dna.affrc.go.jp/KAIKObase/, accessed on 1 March 2021) localized the transgenes to distinct chromosomes.

### 4.4. SDS-PAGE and Western Blotting

Proteins were extracted from cocoon layers according to a previously described method [29], with some modifications. In brief, the cocoon layers were ground into powder using a tissue homogenizer (Tissuelyser-24, Shanghai Jingxin Company, Shanghai, China) and dissolved in SDS buffer (1:20, *wet/vol*) at 37 °C for a couple of hours. Supernatants were collected by centrifugation at 15,000 rpm for 10 min. Each sample was mixed with electrophoresis buffer, incubated at 100 °C for 5 min, then loaded onto SDS-PAGE gels (Sangon, Shanghai, China). Proteins were visualized by staining with Coomassie brilliant blue R-250 or a Fast Silver Stain kit (Beyotime, Shanghai, China). Western blotting was performed using polyclonal antibodies against EGF (ab9695; Abcam, Cambridge, UK) or an anti-P25 antibody (Genscript, Nanjing, China) as the primary antibody and goat anti-rabbit IgG H&L (HRP) (ab205718; Abcam, Cambridge, UK) as the secondary antibody. Band intensities were quantitated using ImageJ software (https://imagej.nih.gov/ij/, accessed on 1 March 2021).

### 4.5. Preparation of Transgenic Silkworms with a P25 Gene Knockout Background

In our previous study (unpublished), a 1-bp deletion in the first exon of the *P25* gene in the diapausing *Bombyx mori* strain *Qiufeng* was generated using a CRISPR/Cas9 system, causing a frameshift mutation in the *P25* gene. As a result, the protein translation terminated early, resulting in the loss of the endogenous P25 protein. The homozygous *P25* gene mutant described above was named P25-D1^−/−^. To prepare transgenic silkworms with this *P25* gene knockout genetic background, the following cross-strategy was used. Transgene detection was performed by screening for marker gene (IE1-DsRed) fluorescence, using a fluorescence microscope as described above. First, one transgenic lineage was selected to mate with the non-transgenic *Qiufeng*. Positive transgenic individuals carrying a single insertion of the hEGF gene were screened for DsRed expression at the generation 1 (G1) larval stage. Transgenic G1 individuals were reared and crossed with homozygous P25-D1^−/−^ individuals to generate a G2 cohort that was P25-D1^+/−^. Transgenic G2 individuals were found by screening larvae for DsRed expression. Both positive and negative transgenic larvae were reared and sib-mated in G2. After screening for DsRed expression at the larval stage, positive transgenic individuals with a character segregation of P25 were expected to yield a 1:2:1 genotype ratio in G3 according to the Mendelian inheritance. Each positive transgenic individual was reared to pupa stage. Genomic DNA was extracted from each silkworm’s exuviation, and PCR amplification was conducted using primers P25-F2 and P25-R2 (Table 2) to detect the genotype of the *P25* gene. Transgenic silkworms with a P25-D1^−/−^ genetic background were identified based on the PCR product sequencing results.

### 4.6. Observation and FTIR Microspectroscopy Analysis of Cocoon Silks

The surfaces of cocoon silks were observed using a digital microscope (VHX-600, Keyence, Osaka, Japan) at 1000-multiple magnification. FTIR microspectroscopy was used to analyze the secondary structure of the silks. A mixture of 2-mg silk powder with 200 mg of potassium bromide (KBr) was made into tablets for analysis using an instrument FTIR-8400S (Shimadzu, Japan). FTIR microspectra were recorded in the range 400–4000 cm^−1^ at a resolution of 4 cm^−1^ with 50 scans. Then, the FTIR spectra were collected to analyze the amide I band (1620–1700 cm^−1^) by Omnic and Origin 9.1 software.

### 4.7. Statistical Data Analysis

Statistical analyses were performed using Student’s *t*-tests. The levels of statistically significantly differences were set at * *p* < 0.05 and ** *p* < 0.01. All data were reported as means ± SEM.

## Figures and Tables

**Table 1:** Outcome of the transgenesis.

Silkworm Strain	Microinjected Embryos	Hatched Embryos (%)	Positive G1 Broods (%)
*Lan 10*	800	103 (12.88)	5 (14.29)

**Table 2:** List of the primer sequences.

Primer	Sequence (5′-3′)
pBacL1-F	GACAAGCACGCCTCAGCC
pBacL1-R	TGAGTCAAAATGACGCATGATTATC
pBacL2-F	GCTCCAAGCGGCGACTG
pBacL2-R	GGGATGTTCTTTAGACGATGAGC
pBacR1-F	TCTGTATATCGAGGTTTATTTA
pBacR1-R	CCGATAAAAACACATGC
pBacR2-F	ACTCAAAATTTCTTCTAAAGTAACAA
pBacR2-R	CTTTAACGTACGTCACAATATG
P25-F2	CGAGGAGAACATTTTGCGCCTTAGA
P25-R2	AACAGTGTTGCCTGATGAGGATGTC

## Data Availability

Data are contained within the article. The data presented in this study are available in the insert article here.
